# Urinary Microbiome Characteristics in Female Patients with Acute Uncomplicated Cystitis and Recurrent Cystitis

**DOI:** 10.3390/jcm10051097

**Published:** 2021-03-05

**Authors:** Jeong-Ju Yoo, Hee Bong Shin, Ju Sun Song, Minjung Kim, Jina Yun, Zisun Kim, Yoo Min Lee, Sang Wook Lee, Kwang Woo Lee, Woong bin Kim, Chang Beom Ryu, Sung-Woo Park, Seong Kyu Park, Ho-Yeon Song, Young Ho Kim, on the behalf of SMS (Soonchunhyang microbiome multi-disciplinary study group)

**Affiliations:** 1 Department of Internal Medicine, Soonchunhyang University Bucheon Hospital, Bucheon 14584, Korea; puby17@naver.com (J.-J.Y.); 19983233@schmc.ac.kr (J.Y.); ryuchb@schmc.ac.kr (C.B.R.); swpark@schmc.ac.kr (S.-W.P.); skpark@schmc.ac.kr (S.K.P.); 2 Department of Laboratory Medicine and Genetics, Soonchunhyang University Bucheon Hospital, Bucheon 14584, Korea; shinhb@schmc.ac.kr; 3 GC Genome, Green Cross Laboratories, Department of Laboratory Medicine, Yongin 16924, Korea; sjusun277@gmail.com (J.S.S.); minjung@gccorp.com (M.K.); 4 Department of Surgery, Soonchunhyang University Bucheon Hospital, Bucheon 14584, Korea; zskim@schmc.ac.kr; 5 Department of Pediatrics, Soonchunhyang University Bucheon Hospital, Bucheon 14584, Korea; flana@schmc.ac.kr; 6 Department of Urology, Soonchunhyang University Bucheon Hospital, Bucheon 14584, Korea; bartol@schmc.ac.kr (S.W.L.); urolkw@schmc.ac.kr (K.W.L.); woongbins@schmc.ac.kr (W.b.K.); 7 Department of Microbiology and Immunology, School of Medicine, Soonchunhyang University, Cheonan 31151, Korea; songmic@schmc.ac.kr

**Keywords:** 16S rRNA next-generation sequencing, cystitis, urinary tract infection, microbiome

## Abstract

Traditionally, the diagnostic mainstay of recurrent urinary tract infection has been urinary culture. However, the causative uropathogen of recurrent cystitis has not been well established. Urine DNA next-generation sequencing (NGS) can provide additional information on these infections. Herein, we compared urine NGS results and urine cultures in patients with acute uncomplicated cystitis (AUC) and recurrent cystitis (RC), and evaluated the difference in microbiome patterns in the NGS results. Patients who underwent urine culture and NGS due to AUC or RC were retrospectively reviewed. All urine samples were collected via a transurethral catheter and studied utilizing a type of NGS called 16S ribosomal RNA gene amplification and sequencing. The sensitivity of urine NGS was significantly higher than that of conventional urine culture (69.0% vs. 16.7%, *p* < 0.05). The detection rate of urine NGS was slightly lower in the RC group than in the AUC group (67.7% vs. 72.7%). Microbiome diversity was significantly higher in the RC group compared to the AUC group (*p* = 0.007), and the microbiome composition was significantly different between the AUC and RC groups. In the urine NGS results, *Pseudomonas, Acinetobacter*, and Enterobacteriaceae were found in the AUC group, and *Sphingomonas*, *Staphylococcus, Streptococcus,* and *Rothia* spp. were detected in the RC group. Urine NGS can significantly increase the diagnostic sensitivity compared to traditional urine culture methods, especially in RC patients. AUC and RC patients had significant differences in bacterial diversity and patterns. Therefore, recurrent cystitis might be approached from a different perspective.

## 1. Introduction

Urinary tract infection (UTI) is among the most common bacterial diseases, affecting more than 1.5 million people per year [1]. The resulting socioeconomic costs are estimated at about 3.5 billion dollars per year in the United States alone [2,3].

Cystitis is usually clinically diagnosed when the patient has subjective symptoms (e.g., dysuria, frequency, urgency, hematuria, back pain, and nocturia), pyuria in urinalysis, or the isolation of uropathogens on conventional urine culture. If cystitis occurs once, it tends to recur in 50% of the patients within one year [2,4]. However, the conventional urine culture has low sensitivity, so the rate of positive urine cultures in patients with acute cystitis is only 60% [5]. In addition, 75% of the patients of clinical practice with recurrent cystitis are taking empirical antibiotics without undergoing tests, such as urinalysis or urine culture, so the sensitivity of subsequent urine culture tests decreases further [6]. Inappropriate antibiotic administration in recurrent cystitis can worsen the subjective symptoms of the patient and increase the expression of drug-resistant bacteria. However, the causative agent of recurrent cystitis is not well established when using the results of a traditional urine culture, and there is no standard treatment for recurrent cystitis, especially if the urine culture test is negative [7].

In the past, there was a misconception that urine is sterile in healthy subjects, so microbiome studies in the urinary system started relatively late compared to those of other organs [8]. However, as studies of next-generation sequencing (NGS) have been actively conducted recently, bacteria have been identified even in the urine of healthy people, and the urinary system is attracting attention as a reservoir of a new microbiome [9,10,11,12,13,14,15]. The microbiome has been associated with a spectrum of urological diseases, from acute infection to cancer [16]. Among them, cystitis is thought to be the first condition to receive help through microbiome analysis such as NGS. In the past, the diagnosis of a UTI depended on a urine culture to identify aerobic or facultative anaerobic uropathogens. However, this method had limitations because the identification of anaerobic bacteria was difficult, and uncertainty existed over whether all the bacteria were identified [11,17]. Recently, the NGS method has overcome these limitations, and currently, bacteria in the human urinary tract have been gradually revealed [18].

The aim of this study was to identify the clinical utility of urine NGS in acute uncomplicated cystitis and chronic recurrent cystitis and identify the differences in the microbiome patterns of each group by NGS.

## 2. Materials and Methods

### 2.1. Patients and Study Protocol

Between March 2020 and August 2020, we collected information on patients who visited a tertiary hospital with typical cystitis symptoms and were diagnosed with acute uncomplicated cystitis or recurrent cystitis. Patients who fulfilled the following inclusion criteria were eligible for this study: (a) patients 20 years of age or older (b) who underwent urinalysis, urine culture, and urine NGS. Patients with anatomical or structural abnormalities such as a stone, pregnancy, or prolonged indwelling catheter were excluded from the study. Finally, we included 42 patients who met the criteria. The clinical and laboratory records of these patients were retrospectively reviewed.

The patients were divided into two groups according to the following definitions. Acute, uncomplicated cystitis was defined as the acute onset of cystitis symptoms (dysuria with frequency, urgency, and/or hematuria, and pyuria) with no prior history of cystitis in the last three years. Recurrent cystitis was defined as dysuria or suprapubic pain and one or more other irritable voiding symptoms (frequency and urgency) among patients with a medical record with pyuria and or urine culture who had acute cystitis twice in the last 6 months and 3 times a year [19].

The study protocol was approved by the Institutional Review Board of our hospital (IRB number SCHBC 2020-11-020). The study protocol conformed to the ethical guidelines of the World Medical Association Declaration of Helsinki.

### 2.2. Urine Culture

All urine samples were collected via a transurethral catheter from patients with suspected cases of cystitis. A loopful of well-mixed urine (0.001 mL) was inoculated onto a blood agar plate and MacConkey agar (Asan Medical, Seoul, Korea) [20,21,22]. The plates were incubated in a 5% CO_2_ incubator overnight at 35 to 37 °C. After 24 and 48 h of incubation, the culture plates were examined, and bacterial growth of ≥10^3^ CFU/mL was considered a significant cut off for bacteriuria [22]. Bacterial identification was made by matrix-assisted laser desorption ionization-time of flight mass spectrometry (ASTA MicroIDSys, Suwon, Korea).

### 2.3. Sample Collection, Transport, and DNA Extraction

The amount of urine required for NGS has not yet been established; however, many studies have reported that at least 30–50 mL has proven to be successful for studies of the bladder and genitourinary microbiota [14,23,24,25]. Therefore, in this study, we used 50 mL urine samples for NGS. Upon collection, samples were immediately sent to the lab to be stored at −20 °C [26]. Within 1–2 days, samples were sent frozen with dry ice in the genetic lab. DNA was extracted with the Chemagic DNA Stool Kit (PerkinElmer Inc., Waltham, MA, USA) according to the manufacturer’s recommendations with pretreatment and modification. The urine specimens (50 mL) were centrifuged at 3000 rpm for 15 min, and the urinary pellets were washed twice with a 10-fold volume of phosphate-buffered saline (PBS). The washed pellets were resuspended in 700 uL of lysis buffer, and this suspension was then added to a silica bead tube. For cell lysis, the bead-suspension mix was vortexed at maximum speed for 5 min. After centrifugation, the supernatant was further subjected to thermal disruption, proteinase K digestion, and bead-binding and elution according to the manufacturer’s instruction. The DNA concentration was determined fluorometrically on the Qubit^®^ 3.0 Fluorometer (Thermo Fisher Scientific, Waltham, MA, USA) using the Qubit^TM^ dsDNA HS Assay Kit.

### 2.4. 16S rRNA Amplicon Sequencing and Bioinformatics Analysis

Prepared DNA was used for 16S library construction using the NEXTflex 16S V4 Amplicon-Seq (BioO Scientific, Austin, TX, USA), and the resulting library was sequenced using the Illumina MiSeq Reagent Kit v2 (500 cycles) following the manufacturer’s protocol. To check the contamination of the DNA extraction process, 16S qPCR analysis was performed on negative controls and urine samples at the time of test set up. The negative controls included a pre-processing control, a lysis buffer control, and a binding buffer control, and all these negative controls had a concentration of 16S at least 10 times lower than the urine samples and were found to be not significantly contaminated compared to urine samples.

We used QIIME 2 to analyze the 16S sequence data. Demultiplexed and primer-trimmed data were quality-filtered and denoised using DADA2 [27,28]. Amplicon sequence variants (ASVs) with fewer than 10 reads or present in only a single sample were removed, and taxonomy was assigned to each ASV using the naive Bayes machine-learning taxonomy classifiers in the q2-feature-classifier against the NCBI refseq database with taxonomic weight assembly using q2-clawback [29,30].

### 2.5. Classification and Definition of Uropathogens

Based on previous reports, we classified the bacteria detected in the urine culture test and urine NGS into three groups of 18–21; (1) definite uropathogens: bacteria already established as uropathogens by conventional diagnostic methods; (2) bacteria less likely to be a uropathogen: bacteria isolated from the urine of healthy men and women, but rarely from the urine of affected individuals; and (3) possible uropathogens: bacteria not included in groups 1 or 2. Groups 1 and 3 were regarded as uropathogens in this study. Detailed information on the bacteria is presented in the Supplementary Data.

### 2.6. Statistical Analysis

For alpha diversity, the sample data were rarefied, and the Shannon indexes, observed operational taxonomic unit (OTUs) (richness), and Simpson’s evenness were calculated. The non-parametric statistical Kruskal–Wallis test was used to test for group differences. To perform beta diversity analysis, we used principal coordinates analysis (PCoA) with weighted Unifrac distances. Permutational multivariate analysis of variance (PERMANOVA) [31]. Implemented in the adonis function of the R/vegan package (v2.5–2) was then performed to identify group differences based on the weighted UniFrac distances. Differentially abundant taxa were visualized using heatmaps with log2-transformed relative abundances and hierarchically clustered based on Bray–Curtis distance. To further explore key genera that may have contributed to the observed differences in the microbial communities, linear discriminant analysis (LDA) effect size (LEfSe) analysis was performed (http://huttenhower.sph.harvard.edu/galaxy, access date: 27 October 2020) to estimate the effect size of differentially abundant features with biological consistency and statistical significance. Herein, the α value for the statistical test was set at 0.05, and the threshold LDA score for discriminative features was set at more than 3.0.

## 3. Results

### 3.1. Baseline Characteristics

The characteristics of the patients are presented in Table 1. The patients were all female, and the mean age was 54.1 ± 12.7 years. The mean age of the recurrent cystitis group was 55.1 years old, which was higher than that of the acute uncomplicated cystitis group, but it was not statistically significant. There were no differences between the groups in terms of previous pregnancy experience and menopause. Twenty-three (54.7%) patients took antibiotics prior to visiting our hospital. The pyuria or bacteriuria urinalysis results were not different between the groups.

### 3.2. Comparison of the Sensitivity of Conventional Urine Culture and Urine NGS

Next, we compared the sensitivity of conventional urine culture with urine NGS. A schematic table of the sensitivity of each group is presented in Table 2. In the urine cultures, bacteria were detected in seven (16.7%) of 42 patients. In urine NGS, bacteria were detected in 29 of 42 patients (69.0%), which was significantly higher than that of the urine cultures (*p* < 0.05). The sensitivity superiority of urine NGS was the same in both the acute uncomplicated cystitis and recurrent cystitis groups.

However, there was a slight difference in the sensitivity of urine NGS between the two groups. The detection rate of urine NGS in uncomplicated cystitis was 72.7% in the acute uncomplicated cystitis group and 67.7% in the recurrent cystitis group, which was slightly lower (*p* = 0.094). In one of the 42 patients, there was a discrepancy between the urine culture test results and the NGS results. The patient belonged to the recurrent cystitis group. *Enterococcus faecalis* was detected in the urine culture, whereas predominantly *Escherichia*/*Shigella* were detected by urine NGS. The raw data of the urine culture and urine NGS are presented in the Supplementary Tables (acute uncomplicated cystitis, Supplementary Table S1; recurrent cystitis, Supplementary Table S2).

### 3.3. Microbiome Diversity of Acute Uncomplicated Cystitis and Recurrent Cystitis

**Figure 1:**
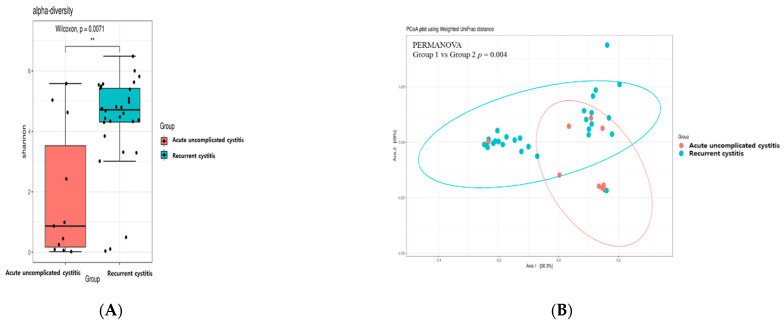
Diversity analysis of acute uncomplicated cystitis and recurrent cystitis, (**A**) Pairwise alpha diversity comparisons of urine microbiota between the two cystitis groups. Boxplot of the Shannon index shows significant differences between the two types of cystitis (** *p* < 0.01), (**B**) Principal coordinate analysis of the urine microbiota based on weighted UniFrac distances between the two cystitis groups shows significant differences in microbial composition.

Alpha diversity analysis was used to determine the distribution of various microorganisms present in one sample. Figure 1A represents the alpha diversity of each group. The microbiome diversity measured by Shannon indexes was significantly higher in the recurrent cystitis group compared to the acute uncomplicated cystitis group (*p* = 0.007). To determine whether the Shannon index value increased in richness and evenness, observed OTUs and Simpson’s evenness analysis were additionally performed, respectively (Supplementary Figure S1). Richness was significantly increased in the recurrent cystitis group. Evenness was also increased in the recurrent cystitis group, but it was not statistically significant.

Whether the microbial community was different between the two cystitis groups was represented by beta diversity. In this study, we used weighted UniFrac distances to calculate beta diversity (Figure 1B). The composition of the microbiome was significantly different between the two groups (*p* = 0.004).

### 3.4. Microbiome Composition of Acute Uncomplicated Cystitis and Recurrent Cystitis

**Figure 2:**
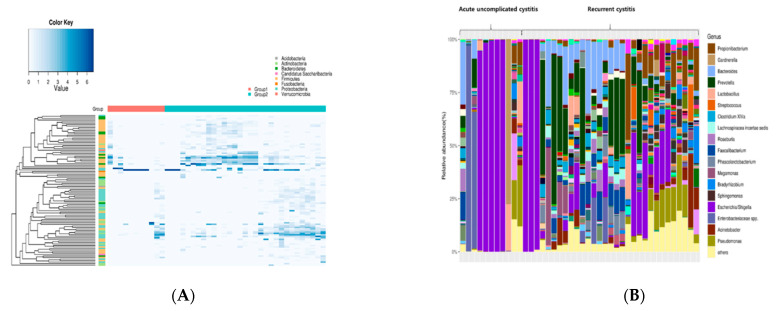
A heatmap showing bacterial genera differentially abundant between the two cystitis groups, (**A**) The columns represent the samples, and the rows represent the top 100 genera based on overall abundance. The value in the heatmap represents the log2-normalized number of sequencing reads, with increasing shades of blue representing greater relative abundance, (**B**) The stacked bar plot shows the relative abundance of genera in each sample sorted by the order in the heatmap (left). Genera with an overall abundance of less than 1% were summed into “Others.”.

Next, we analyzed how the specific bacterial genera differed according to the group. Figure 2 shows the relative abundance of the top 100 genera as a heatmap. In acute uncomplicated cystitis, a single strain tended to be detected predominantly (e.g., Enterobacteriaceae or especially *Escherichia* species). In contrast, in recurrent cystitis, various species other than Enterobacteriaceae were detected. The heatmap showing the differential abundance of bacterial taxa ordered by fold-change is shown in Supplementary Figure S2.

**Figure 3:**
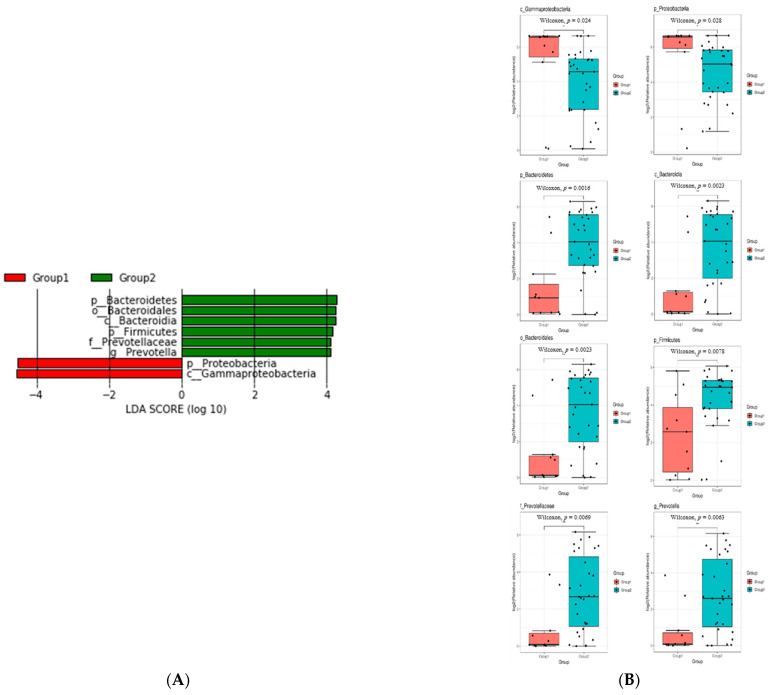
Comparison of bacterial taxa between acute uncomplicated cystitis and recurrent cystitis, (**A**) LEfSe analysis showing the bacterial taxa that were significantly different in abundance between the two cystitis groups. The taxa enriched in the acute uncomplicated cystitis group are shown in red with negative linear discriminant analysis (LDA) scores, and the recurrent cystitis group is shown in green with positive LDA scores. Only taxa passing the LDA threshold value of >2.0 are shown, (**B**) Boxplots showing the log2-transformed relative abundance of eight genera with LDAs above the significant threshold of 3 based on LefSe analysis between the two cystitis groups (* *p* < 0.05, ** *p* < 0.01).

Finally, we analyzed the results according to the ratio of each species detected in each group (Figure 3). Phylum Proteobacteria and Class Gammaproteobacteria were more frequently detected in the acute uncomplicated cystitis group than in the recurrent cystitis group. In contrast, in the recurrent cystitis group, the following organisms were detected at a higher rate compared to acute uncomplicated cystitis: Phylum Bacteroidetes, Class Bacteroidia, Order Bacteroidales, Family Prevotellaceae, and Phylum Firmicutes.

### 3.5. Possible Uropathogens of Acute Uncomplicated Cystitis and Recurrent Cystitis

**Figure 4:**
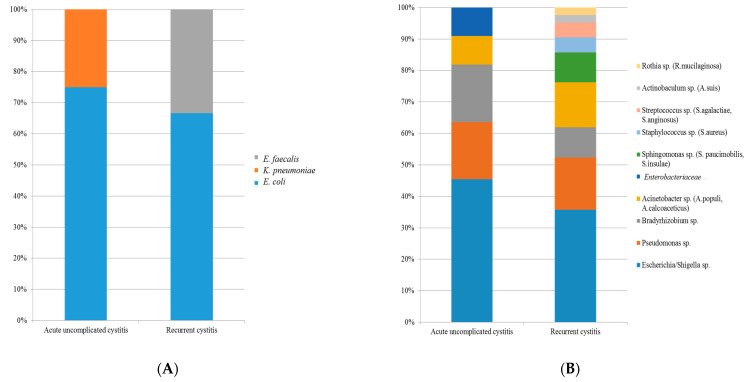
Comparison of frequently encountered uropathogens in acute uncomplicated cystitis and recurrent cystitis, (**A**) Conventional urine culture, (**B**) Urine NGS.

Based on previous reports, we summarized the possible uropathogens found by urine culture tests and urine NGS (Table 3, Figure 4). In the urine culture, *Escherichia coli* was the main detected uropathogen in both the acute uncomplicated cystitis and recurrent cystitis groups (Table 3, Figure 4A). In urine NGS, in addition to *E. coli, Pseudomonas, Acinetobacter, Bradyrhizobium*, and Enterobacteriaceae were additionally found in the acute uncomplicated cystitis group (Table 3, Figure 4B). In the recurrent cystitis group, *Sphingomonas, Staphylococcus, Streptococcus, Rothia*, and others were further identified by urine NGS (Table 3).

## 4. Discussion

In this study, we found that urine NGS was more sensitive than conventional urine culture in both acute uncomplicated cystitis and recurrent cystitis, the sensitivity of urine NGS was lower in recurrent cystitis than in acute uncomplicated cystitis, and acute cystitis and recurrent cystitis had significant differences in bacterial diversity and microbiome patterns.

Our first finding was the superior sensitivity of urine NGS compared to that of urine cultures, which is consistent with existing studies [11,15,32,33]. In previous studies, the false-negative rate of conventional urine culture tests was reported to be as high as 70–90%, while that of urine NGS was reported to be lower than 20%, similar to our study [11,33,34]. In particular, NGS is highly sensitive to atypical bacteria, anaerobes, or urinary tract infections due to multiple species [34]. Furthermore, the sensitivity of NGS is not significantly affected by antibiotic use [35], so it is likely to be useful especially in tertiary hospitals where patients are referred after taking antibiotics in the first and second institutions. In addition, urine NGS does not require time to culture the bacteria, so the time required for testing can be reduced from 3 to 4 days (conventional culture) to within 24 h, [35] helping with rapid clinical decision making and treatment.

The second finding of our study was that acute uncomplicated cystitis and recurrent cystitis had completely different microbiome diversity and patterns, and the two conditions should be approached from different perspectives. An increase in diversity was only observed in the recurrent cystitis group. Previously, the diversity of microbiome in each organ was reported to be associated with various disease manifestations. It was reported that the more bacteria in the vagina and fewer bacteria in the colon, the higher the likelihood of disease occurrence [36,37]. In the urinary system, four studies related to microbiome diversity have been reported [8,38,39,40]. First, Horwitz et al [38] reported that patients with reduced microbiome diversities were more likely to develop urinary tract infections after an indwelling catheter, contrary to our study. In contrast, Swamy et al [40] reported that there was no significant difference in microbiome diversity between patients with asymptomatic pyuria and neurogenic bladder. Our study included patients with typical cystitis symptoms and recurrent cystitis, whereas the previously mentioned two studies included asymptomatic patients. Finally, reports by Kramer et al [39] and Thomas-White et al [8] reported increased diversity in chronic kidney disease and overactive bladder, respectively. To the best of our knowledge, our study was the first to demonstrate that increased microbiome diversity was associated with recurrent cystitis.

Another finding of our study is that the urine WBCs per HPF are very low in the recurrent cystitis. No definition of recurrent UTI is universally accepted, but it is commonly defined as at least two episodes of symptomatic infection (dysuria, frequency, urgency, suprapubic pain, or hematuria), with pyuria or positive bacterial culture, in the past 6 months or three infections in the past 12 months.^19^ In our study, the same diagnostic criteria were applied for recurrent cystitis. In real practice, this definition is often used, but patients who comply with this criterion often do not show high urine WBCs. Because the symptoms of patients and laboratory results do not always match, we tried to accurately evaluate them through the 16s RNA sequencing test. Plus, asymptomatic bacteriuria or positive cultures are common at older ages, so it is difficult to use host responses such as WBC 10+ as a criterion for recurrent cystitis.

Taken together, acute uncomplicated cystitis can be regarded as a temporary infection caused by a specific causative organism, but in recurrent cystitis, dysbiosis seems to play a more important role in the pathophysiology [41]. In addition, even within the same recurrent group, there is a possibility that the dysbiosis changes as the condition is prolonged. Therefore, we carefully suggest that recurrent cystitis might be associated with urinary tract dysbiosis, although further research is needed [42].

To relate this argument to treatment, it is better to use antibiotics quickly for acute uncomplicated cystitis, and for recurrent cases, it is better to decide more carefully whether antibiotic use will help or not according to the NGS results. In particular, in the recurrent group, inappropriate antibiotic administration without discriminating the exact causative agent can worsen the dysbiosis. In acute uncomplicated cystitis, a study comparing the treatment approach according to the conventional urine culture results or the urine NGS results was conducted. In that study, the rapid improvement of subjective symptoms was observed in the NGS group [34]. In contrast, Horwitz et al. reported that long-term antibiotic treatment for an average of one year improved subjective symptoms in chronic recurrent cystitis [40]. However, the above study had the drawback that it was conducted only in pyuric patients. Therefore, it is difficult to apply the same treatment to patients who visited a tertiary hospital with only symptoms and negative urinalysis and urine culture results due to prior antibiotic treatment.

Recently, microbiome modulation studies to reduce dysbiosis have also been conducted. Some studies showed that the use of probiotics improved urinary tract infections, but the results are still insufficient [43,44]. In addition, one study reported that recurrent UTIs were improved when fecal transplantation was performed in renal transplant patients [45]. In a recent study, *Lactobacillus* was shown to have a protective effect against uropathogens, but this protective effect was limited only to women [46].

Our study had several advantages. First of all, the entire urine sample was collected via transurethral catheterization. Thus, the probability of post-bladder contamination or possibility that the symptoms are due to urethral dysbiosis or infection could be minimized compared to other studies that collected midstream urine. Second, our study firstly found that microbiome diversity was increased in the recurrent cystitis group. Third, we further discovered many anaerobic bacteria in NGS that were not detected in the conventional culture method, suggesting the potential utility of the NGS method. This could be the basis for the more active use, in clinical practice, of expanded quantitative urinary culture (EQUC), which can encompass anaerobes. Lastly, our study reported the first probable list of the causative agents of recurrent cystitis. This can provide very useful information on hard-to-treat chronic recurrent cystitis in symptomatic patients with negative cultures, which is the most problematic issue in clinical practice. The information provided on the urine microbiome may provide guidance on the appropriate therapy and increase the understanding of the disease pathophysiology of various urological disorders in the future.

Our study had several limitations. First, there was a limitation caused by the NGS method itself. Since catheterized urine samples are typically low biomass, it would have been better to perform replicates of each sample, as reported previously [8,33]. In addition, NGS does not provide information on antibiotic resistance and is more expensive than conventional urine culture. However, in terms of cost-effectiveness, some argue that NGS is realtively cost-effective, considering inappropriate antibiotic use due to the low positive rate of traditional urine cultures, additional tests, and extension of hospital stays [30]. Second, in our study, we did not provide clear criteria for whether all bacteria detected by NGS were pathogens. However, we presented the classification of uropathogen, but this classification is arbitrary. For example, *Staphylococcus saprophyticus* is a definite urinary pathogen, but in our method, detailed discrimination between *Staphylococcus* species is impossible, so there is no way to confirm. Still, we tried to classify it as much as possible, because we thought that this classification method would be helpful in actual clinical practice. Third, we should be aware that all the identified bacteria do need to be treated. While urine NGS additionally found several bacteria that have not been detected before, we should remember that the asymptomatic bladder is not sterile. As mentioned above, urinary tract dysbiosis plays an important role, especially in the case of recurrent cystitis, and microbiome modulation may be a more realistic goal than the eradication of bacteria by traditional antibiotics. Fourth, our study does not include the information of a healthy control. However, in order to compare with a healthy control, various factors (age distribution, dietary pattern, menopause, hormone therapy, or previous antibiotic use history) must be considered, and a relatively large number of patients was required. Therefore, considering the number of patients required for the study and research cost, this study was designed to compare the microbiota of patients with actual disease status rather than to compare with a healthy control. Finally, our study does not mention the ability of EQUC to reveal the urinary microbiome, despite the outstanding performance of EQUC reported in previous studies [11,12,32,33,47]. We could not use the EQUC method in this study because it is not yet an approved medical practice registered by the government in Korea. If EQUC is approved in Korea in the future, further studies on EQUC will be needed, considering the results of studies that reported good performance in combination with 16S sequencing and enhanced urine culture methods.

In conclusion, our study found that urine NGS could significantly increase detection sensitivity compared to traditional urine culture methods, especially in patients with recurrent cystitis. Additionally, since recurrent and acute uncomplicated cystitis have different causative bacteria, it is better to take different clinical approaches to the two conditions. Additional NGS tests can help make quick decisions and clinical improvement in the patients, especially those with recurrent cystitis.

## Figures and Tables

**Table 1:** Baseline characteristics of the patients.

	Total (*n* = 42)	Acute Uncomplicated Cystitis (*n* = 11)	Recurrent Cystitis (*n* = 31)	*p*
Age (years)	54.10 ± 12.69	51.00 ± 10.72	55.19 ± 13.30	0.291
Female	42 (100)	11 (100)	31 (100)	0.999
Prior pregnancy history	39 (90.4)	10 (90.9)	28 (90.3)	0.955
Menopause	27 (64.2)	6 (54.5)	21 (67.7)	0.433
Prior antibiotic use	23 (54.7)	7 (63.6)	16 (51.6)	0.491
Urinalysis				
Urine RBC (per HPF)	1 (0–28)	0 (0–2)	1 (0–28)	0.226
Urine WBC (per HPF)	1 (0–204)	0 (0–90)	1 (0–204)	0.664
Urine bacteria (per HPF)	0 (0–3+)	0 (0–2+)	0 (0–3+)	0.081
Urine leukocytes (per HPF)	0 (0–3+)	0 (0–3+)	0 (0–3+)	0.021
Urine nitrite (per HPF)	0 (0–1)	0 (0–1)	0 (0–1)	0.132

NOTE: The data are presented as the mean ± standard deviation or median (min–max) for continuous variables and *n* (%) for categorical variables. Abbreviations: RBC, red blood cell; WBC, white blood cell; HPF, high-power field.

**Table 2:** Comparison of the detection rate of conventional urine cultures and urine NGS in acute uncomplicated cystitis and recurrent cystitis.

	Number of Patients	Conventional Urine Culture (+)	Urine NGS (+)
Acute uncomplicated cystitis	11	4 (36.4%)	8 (72.7%)
Recurrent cystitis	32	3 (9.3%)	21 (67.7%)
Total	42	7 (16.7%)	29 (69.0%)

NOTE: The data are presented as *n* (%) for categorical variables. Abbreviations: NGS, next-generation sequencing.

**Table 3:** Frequently encountered genera by urine NGS.

Acute Uncomplicated Cystitis	Recurrent Cystitis
*Escherichia/Shigella*	*Escherichia/Shigella* spp.
*Pseudomonas* spp.	*Pseudomonas* spp.
*Bradyrhizobium* spp.	*Acinetobacter* spp.
*Acinetobacter* spp.	***Sphingomonas* spp.**
***Enterobacteriaceae*** ^a^	*Bradyrhizobium* spp.
	***Staphylococcus* spp.**
	***Streptococcus* spp.**
	***Actinobaculum* spp.**
	***Rothia* spp.**

^a^ The strain is identified as Klebsiella pneumoniae in culture. Bacteria found only in the acute uncomplicated cystitis group and the recurrent cystitis group, respectively, are marked in bold. Abbreviations: NGS, next-generation sequencing.

## Data Availability

The datasets generated during and/or analyzed during the current study are available from the corresponding author on reasonable request.

## Supplementary Materials

The following are available online at https://www.mdpi.com/2077-0383/10/5/1097/s1, Supporting Materials and Method, Figure S1: Alpha diversity of (A) richness, and (B) evenness, Figure S2: The heatmap showing the differential abundance of bacterial taxa ordered by fold-change, Table S1: Comparison of conventional urine culture and urine NGS in acute uncomplicated cystitis, Table S2: Comparison of urine culture and urine NGS in recurrent cystitis.
