# Supplementary material for: Urinary Microbiome Characteristics in Female Patients with Acute Uncomplicated Cystitis and Recurrent Cystitis
Source: J Clin Med. 2021 Mar 5;10(5):1097. doi: 10.3390/jcm10051097 (PMC7961880; doi:10.3390/jcm10051097)
Supplement: Supplementary file 1 [file jcm-10-01097-s001.pdf]

Supporting information

The urinary microbiome characteristics in female patients with acute uncomplicated cystitis and recurrent cystitis

Jeong-Ju Yoo[1*], Hee Bong Shin[2*], Ju Sun Song[3], Jina Yun[1], Zisun Kim[4], Yoo Min Lee[5], Sang Wook Lee[6], Kwang Woo Lee[6], Woong bin Kim[6], Chang Beom Ryu[1], Seong Kyu Park[1], Ho-Yeon Song[7], and Young Ho Kim[6], SMMSG (Soonchunhyang microbiome multi-disciplinary study group)

Table of Contents

1. **Supporting Materials and Methods……………………………………..……..……2**
2. **Supplementary Tables…………..……………………………..……..……..……3**
3. **Supplementary Figure………………………………………………..……..……7**
4. **References……………………..…………………………………..……………9**

Supporting Materials and Method

Classification and definition as uropathogen

Based on the previous reports, we classified bacteria detected in urine culture test and urine NGS into 3 groups as follows.[1-4]

i) **Definite uropathogen: bacteria already established as uropathogens in conventional culture method**
Gram-negative bacteria: Enterobacteriaceae [includes *Escherichia coli*, *Klebsiella* spp, *Proteus* spp, others], *Pseudomonas* spp, other nonfermenting gram-negative rods
Gram-positive bacteria: *Enterococcus* spp, *Staphylococcus aureus*, *Staphylococcus saprophyticus*, *Corynebacterium urealyticum*, *Streptococcus agalactiae* (group B streptococci)

ii) **Bacteria less likely to be a uropathogen: bacteria isolated from the urine of healthy men and women, but rarely from the urine of affected individuals**
Genera: *Lactobacillus, Prevotella, Gardnerella, Actinobaculum, Ureaplasma, Mobiluncus, Veillonella, Faecalibacterium, Aerococcus, Dialister, Porphyromonas, Streptococcus, Actinomyces, Allisonella, Sneathia, Megasphaera, Peptostreptococcus, Peptoniphilus, Anaerococcus, Finegoldia*, *Bradyrhizobium,* other

iii) **Possible uropathogen: bacteria not included in i or ii.**

References

1. *Pohl HG, Groah SL, Perez-Losada M, et al. The Urine Microbiome of Healthy Men and Women Differs by Urine Collection Method. International neurourology journal. 2020;24(1):41-51.*
2. *Neugent ML, Hulyalkar NV, Nguyen VH, Zimmern PE, De Nisco NJ. Advances in Understanding the Human Urinary Microbiome and Its Potential Role in Urinary Tract Infection. mBio. 2020;11(2).*
3. *Siddiqui H, Nederbragt AJ, Lagesen K, Jeansson SL, Jakobsen KS. Assessing diversity of the female urine microbiota by high throughput sequencing of 16S rDNA amplicons. BMC microbiology. 2011;11:244.*
4. *Miller JM, Binnicker MJ, Campbell S, et al. A Guide to Utilization of the Microbiology Laboratory for Diagnosis of Infectious Diseases: 2018 Update by the Infectious Diseases Society of America and the American Society for Microbiology. Clin Infect Dis. 2018;67(6):e1-e94.*

Supplementary Table 1. Comparison of conventional urine culture and urine NGS in acute uncomplicated cystitis

Case	Urine culture	Urine NGS
1	*E.coli*	***Escherichia/Shigella*** 98.1%
2	*E.coli*	***Escherichia/Shigella*** 99.8%
3	*Klebsiella*	***Klebsiella***. 96.8%
4	Negative	*Bacteroides* 29.5%<br>*Faecalibacterium* 12.9%<br>*Roseburia* 10.8%<br>*Dialister* 7.1%<br>*Prevotella* 5.7%
5	Negative	***Escherichia/Shigella*** 93.8%
6	Negative	*Enhydrobacter* 21.8%<br>*Pseudomonas* 18.2%<br>*Propionibacterium* 8.1%<br>*Bradyrhizobium* 7.4%<br>***Acinetobacter*** 6.8%
7	Negative	***Escherichia/Shigella*** 99.3%
8	Negative	***Pseudomonas*** 28.3%<br>*Lactobacillus* 19.4%<br>*Propionibacterium* 10.6%
9	Negative	*Prevotella* 13.6%<br>*Bacteroides* 8.8%
10	Negative	*Gardnerella* 77.5%<br>*Lactobacillus* 22.0%
11	E.coli	***Escherichia/Shigella*** 99.4%
Uropathogenic sensitivity	4/11 (36.4%)	9/11 (72.7%)

Uropathogens are highlighted in bold.

Supplementary Table 2. Comparison of urine culture and urine NGS in recurrent cystitis

Case	Urine culture	Urine NGS (Uropathogens are highlighted in bold)
1	Negative	*Bacteroides* 34.2%<br>*Faecalibacterium* 10.2%<br>*Roseburia* 9.6%<br>*Clostridium XlVa* 6.5%<br>*Ruminococcus* 5.2%
2	Negative	*Prevotella 54.2%*<br>*Phascolarctobacterium 8.0%*<br>*Clostridium XlVa 6.2%*
3	Negative	*Prevotella 44.3%*<br>*Megamonas 15.3%*<br>***Acinetobacter*** *12.2%*<br>*Bacteroides 5.2%*
4	Negative	***Escherichia/Shigella*** *21.6%*<br>*Prevotella 13.8%*<br>*Lactobacillus 8.2%*<br>***Acinetobacter*** *7.7%*<br>*Bacteroides 7.5%*
5	Negative	*Prevotella 31.0%*<br>*Bacteroides 12.0%*<br>*Faecalibacterium 10.1%*<br>*Phascolarctobacterium 7.4%*
6	*E.coli*	***Escherichia/Shigella*** *99.8%*
7	Negative	*Bacteroides 22.0%*<br>***Escherichia/Shigella*** *6.6%*<br>*Roseburia 6.5%*<br>*Faecalibacterium 5.6%*<br>*Anaerostipes 5.1%*
8	Negative	*Bacteroides 29.1%*<br>*Faecalibacterium 13.2%*<br>*Roseburia 10.1%*<br>*Dialister 6.9%*<br>*Anaerostipes 5.7%*<br>*Prevotella 5.4%*
9	Negative	***Pseudomonas*** *20.9%*<br>*Streptophyta 13.9%*<br>*Sphingomonas 6.8%*<br>*Bradyrhizobium 5.5%*
10	Negative	***Escherichia/Shigella*** *33.9%*<br>***Pseudomonas*** *15.9%*<br>*Sphingomonas 6.7%*
11	Negative	***Pseudomonas*** *9.4%*<br>*Propionibacterium 8.0%*<br>***Streptococcus*** *7.0%*<br>*Prevotella 5.3%*<br>***Staphylococcus*** *5.2%*
12	Negative	***Escherichia/Shigella*** *16.1%*<br>*Bacteroides 9.5%*<br>*Phascolarctobacterium 9.5%*<br>*Faecalibacterium 6.3%*<br>*Prevotella 5.0%*
13	Negative	*Lactobacillus 14.0%*<br>*Bacteroides 13.5%*<br>***Escherichia/Shigella*** *8.9%*
14	Negative	*Prevotella 70.3%*<br>*Faecalibacterium 6.9%*

		Bacteroides 6.1%
15	Negative	*Bacteroides 27.2%*<br>*Prevotella 21.6%*<br>*Megamonas 12.8%*<br>***Acinetobacter*** *5.4%*
16	Negative	***Acinetobacter*** *32.6%*<br>***Streptococcus*** *16.5%*<br>*Propionibacterium 7.7%*
17	Negative	*Propionibacterium 20.0%*<br>***Bradyrhizobium***. *17.3%*<br>*Enhydrobacter 11.7%*<br>***Acinetobacter*** *10.1%*<br>*Rheinheimera 8.6%*
18	Negative	*Megamonas 46.0%*<br>*Bacteroides 31.1%*<br>*Clostridium XlVa 6.8%*
19	Negative	*Prevotella 38.4%*<br>*Bacteroides 13.8%*<br>*Faecalibacterium 10.8%*<br>*Phascolarctobacterium 8.9%*<br>*Clostridium XlVa 5.3%*
20	Negative	*Prevotella 35.1%*<br>*Bacteroides 12.9%*<br>*Faecalibacterium 11.1%*<br>*Phascolarctobacterium 7.4%*
21	Negative	*Prevotella 36.0%*<br>***Escherichia/Shigella*** *18.5%*
22	Negative	***Escherichia/Shigella*** *44.2%*<br>*Propionibacterium 15.4%*<br>*Prevotella 7.0%*
23	Negative	*Propionibacterium 26.6%*<br>*Lactobacillus 13.9%*
24	E.faecalis	***Escherichia/Shigella*** *99.0%*
25	Negative	***Escherichia/Shigella*** *24.4%*<br>***Pseudomonas*** *17.4%*<br>*Rothia 13.2%*<br>*Propionibacterium 5.0%*
26	Negative	*Prevotella 45.0%*<br>*Faecalibacterium 14.4%*<br>*Alloprevotella 7.4%*
27	Negative	***Escherichia/Shigella*** *21.2%*<br>***Pseudomonas*** *13.5%*<br>*Gardnerella 7.6%*<br>*Propionibacterium 6.8%*<br>***Bradyrhizobium*** *5.6%*
28	Negative	***Pseudomonas*** *15.4%*<br>*Propionibacterium 15.2%*<br>*Prevotella 5.3%*
29	Negative	***Escherichia/Shigella*** *48.5%*<br>***Streptococcus*** *15.2%*<br>*Prevotella 12.7%*
30	Negative	***Pseudomonas*** *14.7%*<br>*Propionibacterium 14.2%*<br>***Staphylococcus*** *6.6%*<br>***Sphingomonas*** *5.9%*
31	E.coli	***Escherichia/Shigella*** *99.9%*
Uropathogenic sensitivity	3/32 (9.3%)	21/32 (67.7%)

Uropathogens are highlighted in bold

Supplementary Figure S1

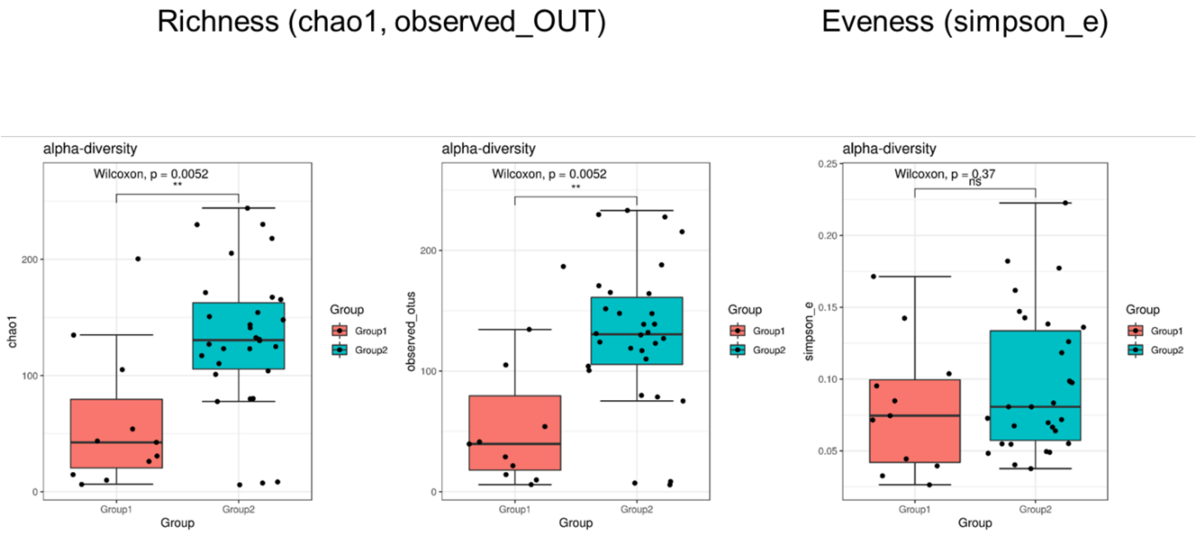

Alpha diversity of (A) richness, and (B) evenness.

Supplementary Figure S2

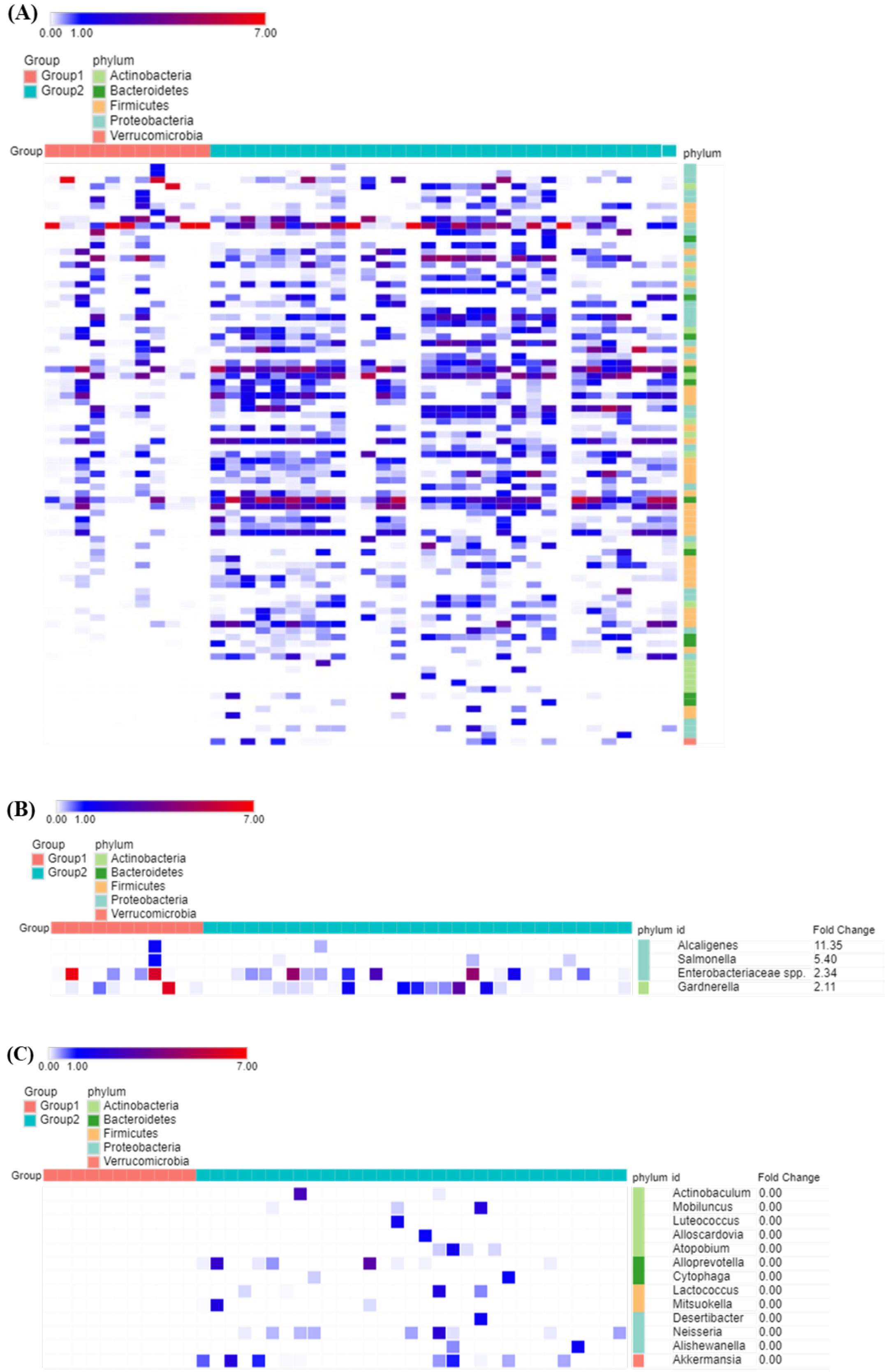

(A) The heatmap shows differential abundance of bacterial taxa at genus level and rows (genus) were ordered by fold change of acute uncomplicated cystitis (comparison A) with respect to recurrent cystitis groups (comparison B). The cystitis groups and comparison groups are indicated by the horizontal colored bar. All taxa present at less than 1% in overall abundance are excluded from the heatmap. The value the in the heat map represent the log2-normalized numbers of sequencing reads, with increasing grades of red representing greater relative abundance. The heatmaps of bacterial taxa with fold change above 2 **(B)** and below than 0, **(C)** respectively, are represented separately in details.
